# Distribution and academic significance of learning approaches among pre-clinical medical students at Trinity School of Medicine, St Vincent and the Grenadines

**DOI:** 10.3352/jeehp.2018.15.9

**Published:** 2018-04-06

**Authors:** Keshab Raj Paudel, Hari Prasad Nepal, Binu Shrestha, Raju Panta, Stephen Toth

**Affiliations:** 1 Department of Pharmacology, Faculty of Pharmacology, Trinity School of Medicine, Ribishi, Rathomill, St. Vincent and the Grenadines; 2 Department of Microbiology, Faculty of Microbiology, Trinity School of Medicine, Ribishi, Rathomill, St. Vincent and the Grenadines; 3 Department of Neuroscience, Faculty of Neuroscience, Trinity School of Medicine, Ribishi, Rathomill, St. Vincent and the Grenadines; 4 Department of Physiology, Faculty of Physiology, Trinity School of Medicine, Ribishi, Rathomill, St. Vincent and the Grenadines; 5 Trinity School of Medicine, Ribishi, Rathomill, St. Vincent and the Grenadines; Hallym University, Korea

**Keywords:** Academic performance, Learning, Medical students, St. Vincent and the Grenadines

## Abstract

**Purpose:**

Different students may adopt different learning approaches: namely, deep and surface. This study aimed to characterize the learning strategies of medical students at Trinity School of Medicine and to explore potential correlations between deep learning approach and the students’ academic scores.

**Methods:**

The study was a questionnaire-based, cross-sectional, observational study. A total of 169 medical students in the basic science years of training were included in the study after giving informed consent. The Biggs’s Revised Two-Factor Study Process Questionnaire in paper form was distributed to subjects from January to November 2017. For statistical analyses, the Student t-test, 1-way analysis of variance followed by the post-hoc t-test, and the Pearson correlation test were used. The Cronbach alpha was used to test the internal consistency of the questionnaire.

**Results:**

Of the 169 subjects, 132 (response rate, 78.1%) completely filled out the questionnaires. The Cronbach alpha value for the items on the questionnaire was 0.8. The score for the deep learning approach was 29.4± 4.6, whereas the score for the surface approach was 24.3± 4.2, which was a significant difference (P< 0.05). A positive correlation was found between the deep learning approach and students’ academic performance (r= 0.197, P< 0.05, df= 130).

**Conclusion:**

Medical students in the basic science years at Trinity School of Medicine adopted the deep learning approach more than the surface approach. Likewise, students who were more inclined towards the deep learning approach scored significantly higher on academic tests.

## Introduction

Students’ learning capability and skills depend on their attitude, aptitude, available facilities (e.g., books, notes, libraries, etc.), curricular strategies, and other aspects of the academic environment. In a medical school, different students may use different approaches when studying, such as the deep and surface approaches [1]. Biggs et al. [2] divided learning approaches into 2 scales (deep and surface), with motive and strategy as subscales in each approach. The deep learning approach is associated with intrinsic motivation, a focus on understanding, a search for logical correlations, and the application of previous knowledge and experience. Conversely, the surface learning approach is associated with an extrinsic pressure to study, difficulty in correlating ideas, and unclear perceptions of the strategy behind studying [3]. Previous studies have shown that students who applied the deep approach to their studies were more motivated and interested in their studies than students who applied the surface approach [1,3]. Furthermore, students using the deep approach had more critical thinking and analytical skills relating to the subject matter. The literature contains inconsistent findings regarding the presence of different learning approaches at different stages of medical education, which may be due to factors such as intrinsic and extrinsic motivation, ethnicity, and grants to pay for studying.

The present study was conducted to investigate the learning approaches of students at Trinity School of Medicine and to correlate learning approaches with academic performance.

## Case presentation

### Ethical statement

Ethical clearance was granted by the Institutional Review Board of Trinity School of Medicine prior to the commencement of the study (no number was provided by the School), and informed consent was given by the students. Students were asked not to disclose their identity in any way, and the confidentiality of their responses was strictly maintained.

### Case

The present study was a questionnaire-based, cross-sectional, observational study that was conducted from January to November 2017. Medical students from the first to fourth terms were included in the study. At Trinity School of Medicine, medical students must complete 5 academic terms (approximately equivalent to 5 semesters) and sit for the United States Medical Licensing Examination (USMLE) Step 1 before they progress to clinical clerkships. Before taking the USMLE Step 1, they must pass the National Board of Medical Examiners (NBME) subject and comprehensive exams. In this study, students’ learning approaches were studied and correlated with their academic scores on the NBME subject exams and cumulative final scores on internal exams (quiz 1, quiz 2, mid-term, and final exams taken at school). A total of 169 questionnaires were distributed and 132 students (response rate of 78.1%) responded. The questionnaires were coded and distributed to the students. Therefore, it was a single-blind process. At Trinity School of Medicine, the faculty conduct all the internal exams and prepare the results. The NBME exam results are sent to Trinity School of Medicine by NBME, and the faculty compile and prepare the results. Hence, faculty have access to all the results and are extremely careful regarding the confidentiality of the information.

The Biggs’s Revised Two-Factor Study Process Questionnaire (R-SPQ-2F) was distributed to the students after obtaining verbal informed consent (Supplement 1). The reliability (internal consistency) coefficient (Cronbach alpha) of the questionnaire was calculated to be 0.8, which suggested good reliability, and based on previous studies, goodness of fit [1]. The Biggs R-SPQ-2F consisted of 20 items, with 10 items related to the deep and the surface approaches, respectively. Of the 10 items for each approach, 5 reflected motives (which referred to why students learn) and the other 5 items reflected the strategy (which referred to how they learn) for a given study approach (deep/surface). The students were asked to provide responses on a 5-point Likert scale (1: never or only rarely true of me; 2: sometimes true of me; 3: true for me about half the time; 4: frequently true for me; and 5: always or almost always true for me).

The responses to the questionnaire were analyzed according to the scoring system provided by Biggs. The scores for the deep and surface approaches were calculated as follows; deep approach score= the sum of all deep motive scores+all deep strategy scores, and surface approach score=the sum of all surface motive scores+all surface strategy scores.

The raw data were compiled and entered into Microsoft Excel 2016 for the analysis (Supplement 2). The Student t-test and 1-way analysis of variance followed by the post-hoc t-test were applied to assess whether differences were statistically significant. The Pearson correlation test was applied to analyze the correlations between learning strategies and the students’ scores. The level of statistical significance was 5% (P= 0.05).

**Fig. 1.**
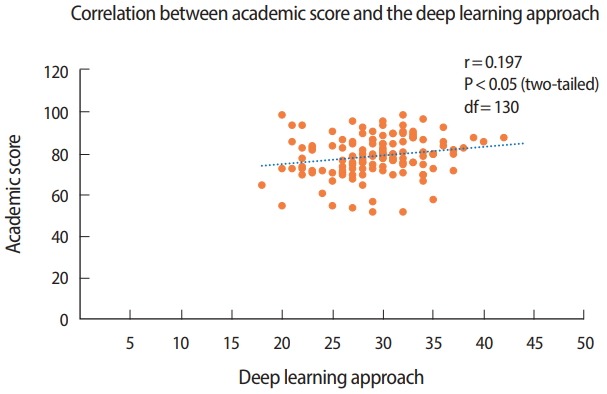
Analysis of the correlation between academic scores and the deep learning approach using the Pearson correlation coefficient (n = 132).

Significant differences were found between the deep and surface learning approaches overall and in terms of motives and strategies (P< 0.01) (Table 1). Table 2 presents a comparison of students in different terms (1, 2, 3, and 4). Fourth term students showed a significantly lower level of deep-learning motives than first- and secondterm students. Similarly, fourth-term students had significantly lower scores for the deep approach than second-term students. Additionally, first-term students utilized the surface approach and strategy significantly more often than second-term students. A positive correlation was found between the deep learning approach and the academic scores of the students (r= 0.197, P< 0.05, df= 130) (Fig. 1).

## Discussion

The present study aimed to characterize the learning approaches of medical students in their preclinical (basic science) years of medical education and to evaluate whether a correlation existed between the deep learning approach and their academic scores. The findings of the study showed that the mean score for the deep learning approach was significantly higher than the mean score for the surface approach (29.4± 4.6 vs. 24.3± 4.2) among the students. The mean scores for the deep learning approach among medical students in previous similar studies were 29.2 at the University of Colombo [4], 33.02 at Harvard Medical School at Beth Israel Deaconess Medical Center [5], and 33.26 at Chitwan Medical College [1]. Similarly, medical students at Trinity School of Medicine were significantly more inclined towards deep motives and strategies than towards surface motives and strategies, and these results are in concordance with the findings of similar studies [1,6]. Medical education results in the training of medical professionals who are highly respected and wellcompensated. Additionally, the medical profession is largely concerned with empathy, efficiency, and successful care. These factors might have contributed to both extrinsic and intrinsic motivations for adopting the deep approach.

Conversely, some inconsistencies were found in terms of which approaches were used in different semesters. Fourth-term students showed less of a deep approach than other term students, in contrast to the findings of previous studies [7,8]. However, this observation agrees with those of Shah et al. [1], Premkumar et al. [9] and Samarakoon et al. [10]. The reason for a greater use of the surface approach by the students in a more advanced academic term (semester) requires further study. However, factors such as approaching board exams, focusing on core areas that are weighed more in the exams, and the lack of time for in-depth study can be hypothesized based on the present findings in terms of factors that promote the surface approach. Although previous studies have suggested that ethnicity may be relevant, as well as cognitive and psychological determinants [11], this study is unable to explain the basis for such results due to its crosssectional nature. Moreover, factors that might have affected the adoption of different learning approaches were not taken into account in this study.

A significant positive correlation was found (r= 0.197, df= 130, P< 0.05; P-value was 0.05 when r was 0.178 at df= 120) between the deep learning approach and academic scores. Students who adopted the deep approach to a greater extent showed better academic performance on standardized tests than students who adhered more to the surface approach. This finding reflects the fact that students who are intrinsically motivated and focused on understanding the course material have a better command of the subject matter and can apply the knowledge in different examination environments, and are more successful academically than students who adopt the surface approach towards their studies.

The limitations of the present study include a small sample size and the fact that it was conducted at a single academic institution. In conclusion, medical students at Trinity School of Medicine were found to adopt the deep learning approach more than the surface approach. Furthermore, students who adopted the deep learning approach performed significantly better on academic examinations, both internal and external. We are planning to use the data of the present study to motivate students to adopt the deep approach so that they may perform better on their exams.

## Figures and Tables

**Table 1.** Comparisons among the variables

Variable	Mean ± standard deviation	P-value (two-tailed)
Deep approach	29.4 ± 4.6	< 0.01
Surface approach	24.3 ± 4.2	
Deep motive	15.6 ± 2.8	< 0.01
Surface motive	12.2 ± 2.3	
Deep strategy	14.2 ± 2.4	< 0.01
Surface strategy	12.2 ± 2.8	

By Student t-test.

**Table 2.** Comparison of learning approaches among students in different terms (n=132)

Term	Deep approach	Deep motive	Deep strategy	Surface approach	Surface motive	Surface strategy
1 (n = 23)	29.9 ± 4.5	16.7 ± 2.6	14.1 ± 2.0	25.7 ± 4.1^a)^	12.7 ± 2.4	13.3 ± 2.7^b)^
2 (n = 30)	30.8 ± 4.9	16.0 ± 2.7	15.1 ± 2.5	23.1 ± 3.8	11.9 ± 2.6	11.2 ± 2.4
3 (n = 51)	29.2 ± 4.5	15.5 ± 2.8	14.1 ± 2.4	24.5 ± 3.9	12.4 ± 2.3	12.2 ± 2.8
4 (n = 28)	27.8 ± 4.3^c)^	14.5 ± 2.8^d),e)^	13.3 ± 2.2	24.1 ± 5.1	11.7 ± 3.0	12.5 ± 3.2

Values are presented as mean±standard deviation. By post-hoc t-test after 1-way analysis of variance. Surface approach: ^a)^P<0.05 between terms 1 and 2; surface strategy: ^b)^P<0.05 between terms 1 and 2; P-values are 2-tailed. Deep approach: ^c)^P<0.05 between terms 4 and 2; deep motive: ^d)^P<0.05 between terms 4 and 2, and ^e)^P<0.01 between terms 4 and 1.

## Supplementary materials

Supplement 1. Biggs’s Revised Two-Factor Study Process Questionnaire (R-SPQ-2F) used in this study [2].

Supplement 2. Data files are available from https://doi.org/10.7910/DVN/BVYQBN

Supplement 3. Audio recording of the abstract.

## References

[1] Shah DK, Yadav RL, Sharma D, Yadav PK, Sapkota NK, Jha RK, Islam MN (2016). Learning approach among health sciences students in a medical college in Nepal: a cross-sectional study. Adv Med Educ Pract.

[2] Biggs J, Kember D, Leung DY (2001). The revised two-factor Study Process Questionnaire: R-SPQ-2F. Br J Educ Psychol.

[3] Mirghani HM, Ezimokhai M, Shaban S, van Berkel HJ (2014). Superficial and deep learning approaches among medical students in an interdisciplinary integrated curriculum. Educ Health (Abingdon).

[4] Subasinghe SD, Wanniachchi DN (2009). Approach to learning and the academic performance of a group of medical students: any correlation. Stud Med J.

[5] Richards JB, Litman J, Roberts DH (2013). Performance characteristics of measurement instruments of epistemic curiosity in third-year medical students. Med Sci Educ.

[6] Brown S, Wakeling L, Naiker M, White S (2014). Approaches to study in undergraduate nursing students in regional Victoria, Australia. Int J Nurs Educ Scholarsh.

[7] Mirghni HO, Elnour MAA (2017). The perceived stress and approach to learning effects on academic performance among Sudanese medical students. Electron Physician.

[8] Sandover S, Jonas-Dwyer D, Marr T (2015). Graduate entry and undergraduate medical students’ study approaches, stress levels and ways of coping: a five year longitudinal study. BMC Med Educ.

[9] Premkumar K, Pahwa P, Banerjee A, Baptiste K, Bhatt H, Lim HJ (2013). Does medical training promote or deter self-directed learning?: a longitudinal mixed-methods study. Acad Med.

[10] Samarakoon L, Fernando T, Rodrigo C (2013). Learning styles and approaches to learning among medical undergraduates and postgraduates. BMC Med Educ.

[11] Shankar PR, Balasubramanium R, Dwivedi NR (2014). Approach to learning of medical students in a Caribbean medical school. Educ Med J.

