# Supplementary material for: Distribution and academic significance of learning approaches among pre-clinical medical students at Trinity School of Medicine, St Vincent and the Grenadines
Source: J Educ Eval Health Prof. 2018 Apr 6;15:9. doi: 10.3352/jeehp.2018.15.9 (PMC5968219; doi:10.3352/jeehp.2018.15.9)
Supplement: Supplementary file 1 — Supplement 1. Biggs’s Revised Two-Factor Study Process Questionnaire (R-SPQ-2F) used in this study [2]. [file jeehp-15-09-suppl.pdf]

Supplement 1. Biggs's Revised Two-Factor Study Process Questionnaire (R-SPQ-2F) [2]

This questionnaire has a number of questions about your attitudes towards your studies and your usual way of studying.

There is no right way of studying. It depends on what suits your own style and the course you are studying. It is accordingly important that you answer each question as honestly as you can. If you think your answer to a question would depend on the subject being studied, give the answer that would apply to the subject(s) most important to you.

Please indicate your answer with the following statements by circling the appropriate number.

Keys:

1 = this item is never or only rarely true of me
2 = this item is sometimes true of me
3 = this item is true of me about half the time
4 = this item is frequently true of me
5 = this item is always or almost always true of me

Please choose the *one* most appropriate response to each question. Fill the oval on the "answer sheet" that best fits your immediate reaction. Do not spend a long time on each item: your first reaction is probably the best one. Please answer each item. Do not worry about projecting a good image. Your answers are CONFIDENTIAL.

Thank you for your cooperation.

1. I find that at times studying gives me a feeling of deep personal satisfaction.
2. I find that I have to do enough work on a topic so that I can form my own conclusions before I am satisfied.
3. My aim is to pass the course while doing as little work as possible.
4. I only study seriously what's given out in class or in the course outlines.
5. I feel that virtually any topic can be highly interesting once I get into it.
6. I find most new topics interesting and often spend extra time trying to obtain more information about them.
7. I do not find my course very interesting so I keep my work to the minimum.
8. I learn some things by rote, going over and over them until I know them by heart even if I do not understand them.
9. I find that studying academic topics can at times be as exciting as a good novel or movie.
10. I test myself on important topics until I understand them completely.
11. I find I can get by in most assessments by memorizing key sections rather than trying to understand them.
12. I generally restrict my study to what is specifically set as I think it is unnecessary to do anything extra.
13. I work hard at my studies because I find the material interesting.
14. I spend a lot of my free time finding out more about interesting topics which have been discussed in different classes.
15. I find it is not helpful to study topics in depth. It confuses and wastes time, when all you need is a passing acquaintance with topics.
16. I believe that lecturers shouldn't expect students to spend significant amounts of time studying material everyone knows won't be examined.
17. I come to most classes with questions in mind that I want answering.
18. I make a point of looking at most of the suggested readings that go with the lectures.
19. I see no point in learning material which is not likely to be in the examination.
20. I find the best way to pass examinations is to try to remember answers to likely questions.

Scoring is in the following cyclical order:

1. Deep motive, 2. deep strategy, 3. surface motive, 4. surface strategy, and 5. etc.

Deep approach score: ∑all deep motive scores+all deep strategy scores

Surface approach score: ∑all surface motive scores+all surface strategy scores
